# A breast cancer prediction model incorporating familial and personal risk factors

**DOI:** 10.1186/1897-4287-10-S2-A29

**Published:** 2012-04-12

**Authors:** Jack Cuzick

**Affiliations:** 1 Centre for Cancer Prevention, Wolfson Institute of Preventive Medicine, Queen Mary University of London, Charterhouse Square, London EC1M 6BQ, UK

## 

Risk factors for breast cancer can be allocated to one of four groups:

1 Family History/genetic

2 Reproductive/hormonal

3 Proliferative benign breast disease

4 Mammographic density

These four factors have now been thoroughly studied and accurate quantitative estimates for the risk are now available for many of them. The most useful summary comes from the Oxford collaboration, which has now produced a series of papers estimating risk for individual factors. Less is known about the possible interaction between these factors and virtually nothing is known about how different factors influence the risk of different types of breast cancer e.g. oestrogen receptor positive versus negative tumours. Risk factors appear to be largely independent and this facilitates building a model to predict risk for individuals. Previous models have focused on either non-genetic factors [5] where important factors relating to genetic risk are not considered, or strictly familial factors in which the modifying effect of other factors is not included [4]. Mammographic density has not been included in any of these models, although it is currently the one risk factor with the largest population attributable risk [2]. Other authors have looked at combined models however [6].

Here we briefly review the main risk factors for breast cancer and describe our own model [7] and a computer programme for synthesizing the factors into an individual risk profile. The model has novel features in terms of combining family history data using segregation analysis with phenotypic factors using the proportional hazards model. Some comparisons between models will be made [1].
